# Draft genome sequence of the almond red leaf blotch pathogen *Polystigma amygdalinum* assembled from infected almond leaves collected in California, USA

**DOI:** 10.1128/mra.00316-26

**Published:** 2026-08-06

**Authors:** Takao Kasuga, Felipe Arredondo, Renaud Travadon, Tawanda E. Maguvu, Alejandro I. Hernandez-Rosas, Florent P. Trouillas

**Affiliations:** 1 USDA-ARS Crops Pathology and Genetics Research Unit 57736 , Davis, California, USA; 2 Department of Plant Pathology, University of California 240443, Davis, California, USA; University of California Riverside, Riverside, California, USA

**Keywords:** *Polystigma amygdalinum*, red leaf blotch, genome assembly

## Abstract

We report a draft genome assembly of *Polystigma amygdalinum*, the causal agent of almond red leaf blotch. DNA extracted from infected leaves was sequenced using PacBio HiFi, and host-derived reads were removed bioinformatically. The 238.7-Mb assembly (90.8% BUSCO completeness) is highly repetitive (82.3%) and unusually large for an ascomycete.

## ANNOUNCEMENT

*Polystigma amygdalinum* is the causal agent of red leaf blotch (RLB), a foliar disease affecting almond production in the Mediterranean basin, the Middle East, and parts of Asia (1). RLB was first detected in California in 2024 (2) and threatens almond production in the state, which supplies 80% of the world’s almonds (3). We generated a genome assembly of *P. amygdalinum*. Infected “Monterey” almond leaves were collected on 26 June 2025 from an orchard in San Joaquin County, California (38°N, 121°W). ITS sequencing showed 100% identity to *Polystigma amygdalinum* isolate TO1 (GenBank accession MH205935.1) (4). Previous studies described *P. amygdalinum* as unculturable (5, 6). Therefore, genomic DNA was extracted directly from 30 infected almond leaves (30%–70% lesion coverage), and host DNA was removed bioinformatically.

High-molecular-weight genomic DNA was extracted at the UC Davis Genome Center Core using a modified CTAB protocol based on Inglis et al. (7), replacing 2-mercaptoethanol with 1% (wt/vol) sodium metabisulfite. A HiFi library was prepared using the SMRTbell Prep Kit 3.0 (Pacific Biosciences). Genomic DNA was sheared to ~14 kb using the PacBio pipette shearing protocol on a CyBio FeliX liquid handler for 200 cycles prior to library preparation. DNA fragments ≤ 10 kb were removed using the LightBench CF system (Yourgene Health). The library (~19.2 kb) was sequenced on PacBio Revio 25M SMRT Cell with SPRQ chemistry and a 30-h movie. HiFi reads were generated using the SMRT Link CCS pipeline. No trimming or quality filtering was performed before assembly. A total of 6,152,879 reads (N50, 13,999 bp) were obtained (Table 1). Default parameters were used unless otherwise noted. Reads were mapped to the almond genome (cv. Nonpareil; GCA_021292205.2) using minimap2 v2.30 (8), and host-derived reads (3,059,033 reads; 40.2 Gb) were removed. The remaining 3,093,846 reads (43.8 Gb) were assembled with Flye v2.9.6 (9) in the –pacbio-hifi mode. Redundant haplotigs were removed using purge_dups v1.2.5 with read-depth thresholds and self-alignment evidence (10). Kraken2 v2.1.3 screening (11) against the NCBI “PlusPFP” database (18 July 2024) identified 37 contigs assigned to *Prunus dulcis* or *Prunus persica*, which were removed. The assembly of 238.7 Mb was unexpectedly large compared with typical filamentous ascomycete genomes of 25–60 Mb (12). Assembly statistics were calculated using seqkit v2.10.1 (13).

**TABLE 1:** Sequencing, assembly, and annotation statistics for the draft genome of *Polystigma amygdalinum*

Parameter	Value
PacBio HiFi sequencing	
Number of reads	6,152,879
Total bases (bp)	84,069,962,314
N50 (bp)	13,999
Reads mapped to the almond genome	3,059,033
Bases mapped to the almond genome (bp)	40,226,196,176
Reads enriched for *P. amygdalinum*	3,093,846
Bases enriched for *P. amygdalinum* (bp)	43,843,766,138
Genome assembly	
Total length (bp)	238,748,157
Number of contigs	159
Maximum contig length (bp)	13,338,927
N50 (bp)	3,768,577
L50	18
GC content (%)	55.5
Average depth of coverage	182×
Genome assessment	
Complete BUSCOs (%)	90.8
Complete and single-copy BUSCOs (%)	89.4
Complete and duplicated BUSCOs (%)	1.4
Fragmented BUSCOs (%)	1.1
Interspersed repeats (%)	82.3
Predicted protein-coding genes	9,060

The assembly comprises 238,748,157 bp in 159 contigs, with a GC content of 55.5% and an N50 of 3,768,577 bp (L50 = 18) with 182× coverage. BUSCO analysis (14) using the sordariomycetes_odb10 data set indicated 90.8% completeness. *De novo* repeat annotations using RepeatModeler v2.0.4 with the -LTRStruct option (15) revealed that interspersed repeats constitute 82.3% of the genome. Protein-coding genes were predicted from the repeat-masked assembly using BRAKER (16), which integrates GeneMark-ET (17) and AUGUSTUS (18). PacBio Iso-Seq transcripts from four developmental stages of almond red leaf blotch lesions (two early stages in May, one intermediate stage in June, and one late stage in August 2025) were used as transcript evidence. Host-derived transcripts were removed, and the remaining transcripts were aligned to the *P. amygdalinum* assembly to guide the prediction of 9,060 genes.

## Data Availability

The genome assembly is available under accession JBUEHE000000000. PacBio HiFi genomic reads are available under SRR38307098. PacBio Iso-Seq transcriptome reads from four developmental stages are available under SRA accession numbers SRR39326659, SRR39326660, SRR39326661, and SRR39326662. All sequence data are associated with BioProject PRJNA1418811.
